# Electronic cigarettes versus nicotine patches for smoking cessation in pregnancy: a randomized controlled trial

**DOI:** 10.1038/s41591-022-01808-0

**Published:** 2022-05-16

**Authors:** Peter Hajek, Dunja Przulj, Francesca Pesola, Chris Griffiths, Robert Walton, Hayden McRobbie, Tim Coleman, Sarah Lewis, Rachel Whitemore, Miranda Clark, Michael Ussher, Lesley Sinclair, Emily Seager, Sue Cooper, Linda Bauld, Felix Naughton, Peter Sasieni, Isaac Manyonda, Katie Myers Smith

**Affiliations:** 1 Wolfson Institute of Population Health, Queen Mary University of London, London, UK; 2 National Drug and Alcohol Research Centre, University of New South Wales, Sydney, New South Wales Australia; 3 Faculty of Medicine and Health Sciences, University of Nottingham, Nottingham, UK; 4 Division of Population Heath Sciences and Education, St Georges, University of London, London, UK; 5 Institute of Social Marketing and Health, University of Stirling, Stirling, UK; 6 Usher Institute and SPECTRUM Consortium, University of Edinburgh, Edinburgh, UK; 7 School of Health Sciences, University of East Anglia, Norwich, UK; 8 The Cancer Research UK and King’s College London Cancer Prevention Trials Unit, King’s College, London, UK; 9 St George’s Healthcare NHS Trust, London, UK

**Keywords:** Medical research, Diseases

## Abstract

Nicotine replacement therapy, in the form of nicotine patches, is commonly offered to pregnant women who smoke to help them to stop smoking, but this approach has limited efficacy in this population. Electronic cigarettes (e-cigarettes) are also used by pregnant women who smoke but their safety and efficacy in pregnancy are unknown. Here, we report the results of a randomized controlled trial in 1,140 participants comparing refillable e-cigarettes with nicotine patches. Pregnant women who smoked were randomized to e-cigarettes (*n* = 569) or nicotine patches (*n* = 571). In the unadjusted analysis of the primary outcome, validated prolonged quit rates at the end of pregnancy in the two study arms were not significantly different (6.8% versus 4.4% in the e-cigarette and patch arms, respectively; relative risk (RR) = 1.55, 95%CI: 0.95–2.53, *P* = 0.08). However, some participants in the nicotine patch group also used e-cigarettes during the study. In a pre-specified sensitivity analysis excluding abstinent participants who used non-allocated products, e-cigarettes were more effective than patches (6.8% versus 3.6%; RR = 1.93, 95%CI: 1.14–3.26, *P* = 0.02). Safety outcomes included adverse events and maternal and birth outcomes. The safety profile was found to be similar for both study products, however, low birthweight (<2,500 g) was less frequent in the e-cigarette arm (14.8% versus 9.6%; RR = 0.65, 95%CI: 0.47–0.90, *P* = 0.01). Other adverse events and birth outcomes were similar in the two study arms. E-cigarettes might help women who are pregnant to stop smoking, and their safety for use in pregnancy is similar to that of nicotine patches. ISRCTN62025374.

## Abstract (web summary)

A randomized controlled trial comparing the efficacy of electronic cigarettes and nicotine patches for smoking cessation in pregnant women found no differences between the interventions. However, electronic cigarettes were found to have some benefit when only the data from women who adhered strictly to the trial protocol were analyzed.

## Main

Smoking in pregnancy increases the risk of adverse birth outcomes such as low birthweight, placental abruption, preterm birth, miscarriage and neonatal or sudden infant death^1–4^. The need to identify stop-smoking interventions that help pregnant women who smoke is made even more urgent by the fact that the link between smoking and socioeconomic disadvantage is particularly strong in women who are pregnant^5^.

To date, two stop-smoking medications have been tested with pregnant women who smoke. Nine placebo-controlled trials evaluated the efficacy of nicotine replacement therapy (NRT)^6–14^ and two trials evaluated bupropion^15,16^, showing only limited effects for NRT and no effect for bupropion^17^. These results could be due to low treatment adherence and, in the case of NRT, also to limited nicotine delivery. Nicotine is metabolized faster in pregnant women who smoke than in women who smoke and are not pregnant, and standard NRT dosing might be too low^8,18–21^.

Electronic cigarettes (e-cigarettes) are devices that deliver nicotine and flavorants in an aerosol that is created by heating propylene glycol and vegetable glycerol^22^. E-cigarettes can be seen as a form of NRT, although they are a consumer rather than pharmaceutical product. E-cigarettes may have several potential advantages over traditional NRT products, such as nicotine patches and gum, in that they enable individuals who smoke to titrate nicotine intake to their needs, select flavors they like, and retain a degree of enjoyment that they previously obtained from smoking^22–26^. E-cigarettes are more popular than traditional NRT products among people who are trying to stop smoking^27,28^, and the first few trials comparing the two treatments in non-pregnant participants suggest that e-cigarette are more effective than NRT for aiding smoking cessation^29,30^. The use of e-cigarettes as a quitting aid has also increased in pregnant women who smoke^31–33^, although the efficacy and safety of such use is unknown.

The use of e-cigarettes in women who are pregnant raises similar concerns about the potential harmful effects of nicotine on the developing fetus to those regarding the use of nicotine patches or chewing gum. The use of medicinal NRT products to help pregnant women to stop smoking is approved in a number of countries because although NRT contains nicotine, tobacco smoke also contains many other toxins with documented teratogenic effects^34–39^. The evidence that nicotine is teratogenic is also only available from animal studies^36^. It is currently not clear whether nicotine has detrimental effects in pregnancy when used at doses consumed by humans who smoke. Two recent reviews concluded that existing data do not provide clear evidence on whether the use of NRT during pregnancy is harmful to the fetus^1,40^. Given that the issue has not been definitively settled, and given that e-cigarette aerosol contains other chemicals in addition to nicotine^41^, objective data on pregnancy outcomes in women who switch from smoking to e-cigarette use are urgently needed.

Here, we compare the efficacy and safety of e-cigarette and NRT patches when used to help pregnant women who smoke to attain prolonged abstinence from smoking in a randomized controlled trial.

## Results

**Fig. 1:**
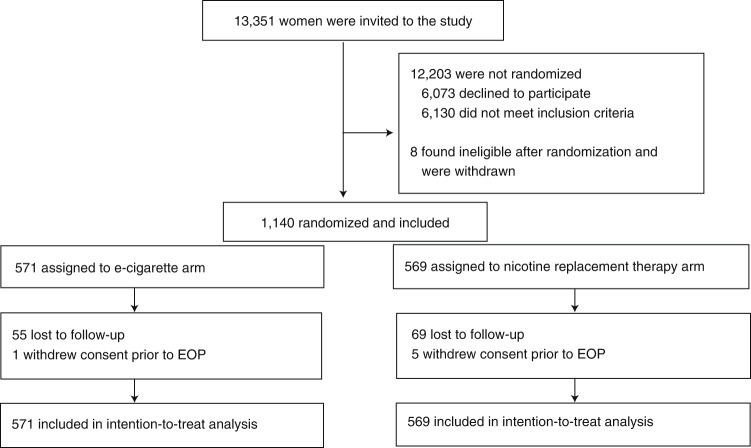
Participants flow. Diagram of the flow of participants through the trial. Participants were recruited from 23 hospital sites in England and from one NHS Stop Smoking Service in Scotland.

Recruitment took place between 11 January 2018 and 7 November 2019. Participants were recruited from 23 hospital sites across England and one National Health Service Stop Smoking Service in Scotland. Participant characteristics are listed in Table 1. Participants had a median age of 27 years, smoked 10 cigarettes per day, and were on average 15.7 weeks pregnant. The profiles of participants in the two study arms were similar. Figure 1 shows the flow of participants through the trial. We were able to establish self-reported smoking status at the end of pregnancy, via direct contact or hospital records, in 531 (93%) and 516 (91%) participants in the e-cigarette and NRT arms, respectively.

**Table 1:** Baseline sample characteristics

	E-cigarettes (*n* = 571)	NRT (*n* = 569)
Age (years), median (IQR)	26.6 (22.5–30.9)	27.3 (23.6–31.1)
Education, *n* (%)		
Primary and secondary school	229 (40.1)	234 (41.1)
Further education	288 (50.4)	273 (48.0)
Higher education	54 (9.5)	62 (10.9)
Employed, *n* (%)	274 (48.0)	257 (45.2)
Ethnicity, *n* (%)		
White British	513 (89.8)	495 (87.0)
Other	58 (10.2)	74 (13.0)
Cigarettes per day, median (IQR)	10 (7–15)	10 (7–15)
FTCD score, mean (s.d.)	4.0 (2.1)	4.3 (2.1)
Cotinine (ng ml^−1^) median (IQR)^a^	111 (75.8–165)	118 (73.9–176)
Lives with person who smokes, *n* (%)	342 (59.9)	328 (57.6)
Past treatment, *n* (%)^b^		
Champix	69 (12.1)	79 (13.9)
NRT	268 (46.9)	273 (48.0)
Zyban	7 (1.2)	5 (0.9)
None	272 (47.6)	267 (46.9)
Tried e-cigarettes in the past, *n* (%)	288 (50.4)	267 (46.9)

FTCD, Fagerstrom test of cigarette dependence.

^a^E-cigarettes, *n* = 529; NRT, *n* = 531 (cotinine at baseline was missing for 80 participants (7.0%), 53 due to insufficient samples and 27 due to loss at the hospital or in the mail).

^b^More than one treatment could be selected.

### Primary outcome

Useable saliva samples were obtained from 108 of 196 self-reported abstinent participants at the end of pregnancy (55.1%; 66 in the e-cigarette arm and 42 in the NRT arm). Of those providing saliva samples, 13 also provided a carbon monoxide reading, while seven participants provided carbon monoxide readings only.

Due to the low return of saliva samples, validated prolonged abstinence rates in the intention-to-treat analysis were low (39 of 571 (6.8%) versus 25 of 569 (4.4%) in the e-cigarette and NRT arms, respectively) and did not differ significantly between the two study arms (Table 2, Bayes factor = 2.69, indicating that the data are insensitive). Pre-specified per-protocol and multiple-imputation analyses yielded similar results, but in the pre-specified sensitivity analysis excluding abstinent participants who regularly used non-allocated products, the difference between the two study arms was significant (39 of 571 (6.8%) versus 20 of 569 (3.6%), Table 2, Bayes factor = 10.0).

**Table 2:** Smoking cessation outcomes

	E-cigarettes (*n* = 571)	NRT (*n* = 569)	RR (95%CI)^a^
Primary outcome			
Validated prolonged abstinence at EOP, *n* (%)	39 (6.8)	25 (4.4)	1.55 (0.95–2.53) *P* = 0.08
Sensitivity analyses			
Per protocol, *n* (%) (*n* = 483 and 382, respectively)	39 (8.1)	23 (6.0)	1.34 (0.82–2.21) *P* = 0.25
Multiple imputation (%)	(9.9)	(7.1)	1.39 (0.90–2.14) *P* = 0.13
Abstinent participants using non-allocated products excluded, *n* (%) (*n* = 571 and 564, respectively)	39 (6.8)	20 (3.6)	**1.93 (1.14–3.26)** ***P*** = **0.02**
Secondary outcomes			
Self-reported abstinence at 4 weeks, *n* (%)	89 (15.6)	61 (10.7)	**1.45 (1.07–1.97)** ***P*** = **0.02**
Self-reported prolonged abstinence at EOP, *n* (%)	63 (11.0)	44 (7.7)	1.43 (0.99–2.06) *P* = 0.06
Validated PP abstinence at EOP, *n* (%)	58 (10.2)	40 (7.0)	1.44 (0.98–2.13) *P* = 0.06
Self-reported PP abstinence at EOP, *n* (%)	118 (20.7)	78 (13.7)	**1.51 (1.16–1.96)** ***P*** = **0.002**
Sensitivity analyses with exclusion of abstinent participants using non-allocated products			
Self-reported abstinence at 4 weeks, *n* (%) (*n* = 570 and 556, respectively)	88 (15.4)	48 (8.6)	**1.79 (1.28**–**2.49)** ***P*** = **0.001**
Self-reported prolonged abstinence at EOP, *n* (%) (*n* = 569 and 556, respectively)	61 (10.7)	31 (5.6)	**1.92 (1.27**–**2.92)** ***P*** = **0.002**
Validated PP abstinence at EOP, *n* (%) (*n* = 569 and 558, respectively)	56 (9.8)	29 (5.2)	**1.89 (1.23**–**2.92)** ***P*** = **0.004**
Self-reported PP abstinence at EOP, *n* (%) (*n* = 565 and 544, respectively)	112 (19.8)	53 (9.7)	**2.03 (1.50**–**2.76)** ***P*** < **0.001**

EOP, end of pregnancy; PP, point prevalence.

^a^Relative risk obtained using a binomial regression with a logarithmic link with two-sided *P* values.

**Bold**, statistically significant results.

### Secondary outcomes

Relative risk (RR) favored the e-cigarette arm to a similar extent for self-reported abstinence (63 of 571 (11.0%) in the e-cigarette arm versus 44 of 569 (7.7%) in the NRT arm; *P* = 0.06) and for validated point prevalence abstinence at the end of pregnancy (58 of 571 (10.2%) in the e-cigarette arm versus 40 of 569 (7.0%) in the NRT arm; *P* = 0.06) (Table 2). Statistical significance was reached, however, only for self-reported abstinence at 4 weeks (89 of 571 (15.6%) in the e-cigarette arm versus 61 of 569 (10.7%) in the NRT arm; RR = 1.45, 95%CI: 1.07–1.97, *P* = 0.02) and for self-reported point prevalence abstinence at the end of pregnancy (118 of 571 (20.7%) in the e-cigarette arm versus 78 of 569 (13.7%) in the NRT arm; RR = 1.51, 95%CI: 1.16–1.96, *P* = 0.002).

In a pre-specified sensitivity analysis we excluded abstinent participants who used non-allocated products. Of the participants who self-reported point prevalence abstinence at the end of pregnancy, six in the e-cigarette arm and 25 in the NRT arm were regularly using non-allocated products (Supplementary Table 1). The differences between the two study arms in this sensitivity analysis were significant for all outcomes: self-reported abstinence at 4 weeks (88 of 570 (15.4%) in the e-cigarette arm versus 48 of 556 (8.6%) in the NRT arm; *P* = 0.001), self-reported prolonged abstinence at the end of pregnancy (61 of 569 (10.7%) in the e-cigarette arm versus 31 of 556 (5.6%) in the NRT arm; *P* = 0.002), validated abstinence at the end of pregnancy (56 of 569 (9.8%) in the e-cigarette arm versus 29 of 558 (5.2%) in the NRT arm; *P* = 0.004) and self-reported point prevalence abstinence at the end of pregnancy (112 of 565 (19.8%) in the e-cigarette arm versus 53 of 544 (9.7%) in the NRT arm; *P* < 0.001) (Table 2).

There was no difference between the study arms in the proportion of non-abstaining women with validated reduction of smoking at the end of pregnancy by at least 50% compared with baseline (12 of 453 (2.7%) in the e-cigarette arm versus 12 of 491 (2.4%) in the NRT arm; *P* = 0.84). Self-reported smoking reduction was significantly more frequent in the e-cigarette arm than in the NRT arm (192 of 453 (42.4%) in the e-cigarette arm versus 166 of 491 (33.8%) in the NRT arm; *P* = 0.007) (Supplementary Table 2).

Table 3 lists the data on treatment adherence in the two study arms. Approximately 30% of participants did not set a target quit date, and this proportion was similar in the two study arms. The uptake of support phone calls was low in both study arms. Product use was initially also low in both study arms but higher in the e-cigarette arm. More participants in both arms used their products during pregnancy, with use higher in the e-cigarette arm, in which one-third of the participants used e-cigarettes at the end of pregnancy.

**Table 3:** Treatment adherence

	E-cigarettes (*n* = 571)	NRT (*n* = 569)	RR (95%CI)^a^
TQD set, *n* (%)	418 (73.2)	394 (69.2)	1.06 (0.98–1.14) *P* = 0.14
Support sessions completed, median (IQR)	1 (0–3)	1 (0–2)	0 (−0.31 to 31)^b^ *P* = 1.00
Allocated product use, *n* (%)			
Did not use allocated product at all	88 (15.4)	184 (32.3)	**0.48 (0.38**–**0.60)** ***P*** **<** **0.001**
Request after the initial 2 week supply	315 (55.2)	207 (36.4)	**1.52 (1.33**–**1.73)** ***P*** **<** **0.001**
Current use at 4 weeks	228 (39.9)	128 (22.5)	**1.78 (1.48**–**2.13)** ***P*** **<** **0.001**
Regular use during study^c^	438 (76.7)	292 (51.3)	**1.49 (1.36**–**1.64)** ***P*** **<** **0.001**
Current use at end of pregnancy	193 (33.8)	32 (5.6)	**6.01 (4.21**–**8.58)** ***P*** **<** **0.001**
Non-allocated product use, *n* (%)			
Current use at 4 weeks	11 (1.9)	56 (9.8)	**0.20 (0.10**–**0.37)** ***P*** **<** **0.001**
Regular use during study^c^	16 (2.8)	101 (17.8)	**0.16 (0.09**–**0.26)** ***P*** **<** **0.001**
Current use at EOP	4 (0.7)	49 (8.6)	**0.08 (0.03**–**0.22)** ***P*** **<** **0.001**

EOP, end of pregnancy; TQD, target quit date.

**Bold**, statistically significant results.

^a^Relative risk obtained using a binomial regression with a logarithmic link with two-sided *P* values.

^b^Median difference (95%CI).

^c^Used for 5+ days during the first 4 weeks or at end of pregnancy, using currently or have used regularly for at least 1 week or occasionally for at least 3 weeks.

Table 3 also lists the data on product use during the study. Regarding the current use of any nicotine product (allocated or unallocated) at the end of pregnancy by participants self-reporting point prevalence abstinence from smoking, 58 of the 118 abstinent participants in the e-cigarette arm (49.2%) reported using a nicotine product (57 allocated and 1 non-allocated) while 15 of the 78 abstinent participants in the NRT arm (19.2%) reported such use (5 allocated, 8 non-allocated and 2 both) (χ^2^(1 d.f.) = 18.0, *P* < 0.001).

Of the 238 participants in the NRT arm who reported using NRT since the last support call, 236 (99.2%) used patches, including 16 who used a combination of patches with other NRT products; one used only an inhaler and one used only mouth spray. Of 351 patch products dispensed by the study team, only 29 (8.3%) were for 10 mg nicotine patches, while the rest were for the 15 mg nicotine patches.

The 344 participants in the e-cigarette arm who used e-cigarettes during at least one of the initial 4 weeks almost exclusively used refillable e-cigarettes (94.2%) (Supplementary Table 3). Most used e-cigarette liquids with a higher nicotine content (11–20 mg ml^−1^) and with tobacco and fruit flavors. For the 244 participants who provided information on their products at 4 weeks and at the end of pregnancy, the nicotine concentration in their e-cigarette liquids decreased significantly over time (Bhapkar χ^2^(2 d.f.) = 32.0, *P* < 0.001).

Safety data were available from 1,110 of the 1,140 women in the study (97.4% of the sample; 556 of 571 in the e-cigarette arm versus 554 of 569 in the NRT arm, 97.4% in each arm). A total of 39 participants (20 in the e-cigarette arm and 19 in the NRT arm) delivered infants at non-study sites and no data were available on birthweight for 10 of them and on gestational age and birthweight for 29. Two women (one in each arm) had an elective termination and were excluded from the analyses. There were 1,095 singleton births and 13 pairs of twins (9 pairs in the e-cigarette arm and 4 pairs in the NRT arm).

Regarding the singleton births, the mean birthweight and rates of adverse birth outcomes were similar in the two study arms, apart from the NRT arm having more infants with low birthweight (52 of 541 (9.6%) in the e-cigarette arm versus 80 of 541 (14.8%) in the NRT arm) (Table 4, Bayes factor = 10.3). The analysis including twins did not change these results (Supplementary Table 4).

**Table 4:** Birth outcomes in the two study arms

	E-cigarettes (*n* = 546)^a,b^	NRT (*n* = 549)^a,b^	RR (95% CI)^c^
Miscarriage, *n* (%)	2 (0.4)	3 (0.6)	0.67 (0.11–4.00) *P* = 0.66
Stillbirth, *n* (%)	2 (0.4)	0 (0)	NC
Neonatal death, *n* (%)	2 (0.4)	3 (0.6)	0.67 (0.11–4.00) *P* = 0.66
Post-neonatal death, *n* (%)	0	3 (0.6)	NC
Maternal death, *n* (%)	0	0	NC
Preterm birth, *n* (%)	46 (8.4)	63 (11.5)	0.73 (0.51–1.05) *P* = 0.09
Low birthweight, *n* (%) (*n* = 541 and 541, respectively)	52 (9.6)	80 (14.8)	**0.65 (0.47–0.90)** ***P*** = **0.01**
NICU admission, *n* (%)	51 (9.3)	46 (8.4)	1.11 (0.76–1.63) *P* = 0.58
Congenital abnormalities, *n* (%)^d^	25 (4.6)	15 (2.7)	1.68 (0.89–3.14) *P* = 0.11
Terminations, *n* (%)			
Due to congenital abnormalities	1 (0.2)	2 (0.4)	1.51 (0.25–9.00) *P* = 0.65
Due to premature rupture of membranes	2 (0.4)	0	NC
Number of women with adverse birth outcomes, *n* (%)	112 (20.5)	119 (21.7)	0.95 (0.75–1.19) *P* = 0.64
Delivery by cesarean section, *n* (%)	131 (24.0)	148 (27.0)	0.89 (0.73–1.09) *P* = 0.26
Gestational age (weeks), mean (s.d.) (*n* = 545 and 547, respectively)	38.4 (3.0)	38.2 (3.1)	0.23 (−0.14 to 0.59)^e^ *P* = 0.22
Birthweight (kg), mean (s.d.) (*n* = 541 and 541, respectively)	3.1 (0.60)	3.1 (0.62)	0.03 (−0.04 to 0.10)^e^ *P* = 0.45

NC, not calculated; NICU, neonatal intensive care unit.

**Bold**, statistically significant results.

^a^Participants are included more than once if they had more than one event.

^b^Singleton births only.

^c^Relative risk obtained using a binomial regression with a logarithmic link with two-sided *P* values.

^d^Two infants in the e-cigarette arm and one in the NRT arm had two congenital abnormalities.

^e^Mean difference (95%CI).

Rates of other adverse events were also similar in the two groups (Supplementary Table 5). Adverse reactions related to the study products consisted primarily of skin irritation and nausea in the NRT arm, and cough and throat irritation in the e-cigarette arm (Supplementary Table 5).

The overall number of serious adverse events and adverse events was 476 in the e-cigarette arm and 479 in the NRT arm. The number of participants with any serious adverse events or adverse events was 285 in the e-cigarette arm and 292 in the NRT arm (RR = 0.97, 95%CI: 0.87–1.09, Supplementary Tables 5 and 6).

## Discussion

In the primary analysis, prolonged validated abstinence rates in the two study arms were not significantly different. Safety outcomes were similar in the two study arms, apart from low birthweight (<2,500 g), which was less frequent in the e-cigarette arm.

Although the two study arms did not differ in the unadjusted primary analysis, in a pre-specified sensitivity analysis excluding abstinent participants who regularly used non-allocated products, e-cigarettes were significantly more effective than NRT. This sensitivity analysis may have overestimated the treatment effect if some ‘switchers’ had succeeded even if they did not use non-allocated products, but other approaches to controlling for different rates of unallocated product use in the two study arms are challenging (Methods).

One limitation of our study is that the biochemical validation of abstinence via posted saliva samples proved challenging. Validation results were available from only about half of the self-reported abstinent participants. Asking women who are in late pregnancy or who are looking after a newborn infant to self-sample and post the samples back generated limited response. Some samples also had an insufficient volume for the analysis and some participants who were abstinent during pregnancy were reached only after delivery, when they had returned to smoking, and therefore validation could not be done. During the follow-up study period, the COVID-19 lockdown further reduced the rate of sample return, although not substantively. These factors resulted in low validated quit rates and reduced the power to detect a difference between the two study arms. Future studies may consider shorter follow-up windows and aim to collect validation samples in person.

Study results might also have been affected by an external event that occurred during the trial. In 2019 there was an outbreak of a lung disease in young vapers in the United States. This was termed ‘e-cigarette or vaping product use-associated lung injury’ (EVALI) and although it was eventually traced to the addition of vitamin E acetate to local illicit marijuana products^42,43^, it was widely reported internationally, including in the United Kingdom, as being related to nicotine vaping^44^. Anecdotal evidence from follow-up calls suggested that the media warning about the dangers of e-cigarettes use led some participants to stop using e-cigarettes and return to smoking.

Treatment adherence and overall abstinence rates were low, as in other studies of smoking cessation in pregnancy^6,9,11,13,14,45,46^. Compared with other individuals who smoke, women who wish to stop but are still smoking at 12 weeks of gestation are likely to have higher nicotine dependence and experience more severe withdrawal symptoms and cravings than other adults^47^. They may also have more uncertain motivation and/or competing priorities. Almost 30% of the participants did not set up a quit date, completion of support sessions was low, and only 40% and 23% used their products for at least 4 weeks in the e-cigarette and NRT arms, respectively. A substantial proportion of participants may thus not have made sufficient use of the support that was offered to benefit from it.

Within the relatively low treatment uptake, the use of e-cigarettes was higher and of longer duration than the use of NRT. This is despite the fact that the cost to participants favored NRT, given that it was provided free of charge, while in the e-cigarette arm the participants had to pay for their own e-cigarette supplies after the initial provision. This advantage, however, could have been mitigated by participants needing a prescription for NRT but not for e-cigarettes. The finding of better use of e-cigarettes tallies with e-cigarettes being a more popular aid to stopping smoking than NRT among people who smoke at the population level^27^. It is worth noting that although the cost to participants was higher for e-cigarettes, the cost to treatment providers was higher for NRT.

There are several other limitations to the generalization of the study findings. Participants may have had different expectations regarding the two study products, although their previous experience with e-cigarettes and NRT was similar (~50% have tried each product previously). We tried to mitigate this potential bias by including only participants who were willing to use either product and by avoiding any indication that one product may be superior to the other in the information to participants, but despite these provisions more participants in the NRT arm never started product use. Engagement with treatment could also affect the response to follow-up calls. More participants in the NRT arm answered the follow-up calls only after delivery, although the difference did not reach statistical significance and the time lapse between delivery and follow-up was shorter in the NRT arm (Supplementary Table 7). Participants received several support calls throughout the study period. Although the completion rate of the calls was low, the results may not be able to be generalized to settings in which no support is available. Regarding the blinding at follow-up calls, different teams conducted the support and follow-up calls but they occasionally covered for each other. A possibility of a re-contact cannot be ruled out, but in a trial with a large sample conducted over a period of more than 2 years, it would be unlikely that researchers would be able to recall participants’ names or their allocation. Participants almost exclusively used refillable e-cigarettes with a maximum of 20 mg/ml nicotine, given that higher nicotine concentrations are banned in the EU. Hence, the results may not be able to be generalized to modern pod e-cigarettes products with higher nicotine delivery. The NRT arm used nicotine patches almost exclusively. In studies that excluded pregnant women who smoked, the combination of patches with other NRT products was shown to be more effective than the use of a single NRT^48^.

Regarding safety outcomes, significantly more infants had low birthweight (<2,500 g) in the NRT arm. This could be a chance finding, but in a previous large study that compared nicotine and placebo patches, the nicotine arm had better birth and infant outcomes than the placebo arm throughout 2 years after delivery^49^. Both findings could be due to a larger reduction in smoking in the study arms with the more favorable safety outcomes. There were more congenital abnormalities in children in the e-cigarette arm, but the difference between the study arms was not significant. The overall incidence of adverse effects in the two study arms was similar. The findings do not suggest that e-cigarette use in pregnancy poses larger risks than the use of NRT, despite the fact that e-cigarettes were more likely to be used and were used for longer periods than NRT.

Adverse reactions linked to the study products consisted primarily of skin irritation and nausea in the NRT arm and throat irritation and cough in the e-cigarette arm. E-cigarettes might have been more acceptable to pregnant women who smoke given that more participants interrupted product use due to adverse reactions in the NRT arm.

The study data may contribute to our understanding of the effects of nicotine on its own in later pregnancy. In animal studies, nicotine dosing in pregnancy generated a range of serious detrimental effects^50–52^ but it is unclear whether this applies to the doses of nicotine and dosing schedules that are used by humans^53^. One report found a higher prevalence of low birthweight and preterm birth in women who used e-cigarettes compared with those who did not, but the sample of women not using e-cigarettes comprised almost exclusively of participants who never smoked, while women who did use e-cigarettes are likely to have smoked at some stage during pregnancy^54^. Another cohort study that compared pregnant women who smoked and who switched completely to e-cigarette use with both those who did not smoke and those who did found that the birthweight of infants of mothers who used e-cigarettes matched that of infants of mothers who did not smoke and was higher than in infants of mothers who did smoke^55^. In this trial, mean birthweight was the same in both study arms, despite the higher use of nicotine products in the e-cigarette arm, and the incidence of low birthweight (<2,500 g) was actually higher in the NRT arm. Nicotine in late pregnancy thus did not seem to have contributed to restricted prenatal growth caused by smoking, but the finding does not encompass nicotine use in early pregnancy, given that all participants were smoking in the first trimester. Nicotine may also pose other risks, and e-cigarettes deliver other chemicals. A cross-sectional cohort study compared Neonatal Behavioral Assessment Scale scores in women who did not smoke, women who smoked, and those who switched to e-cigarettes, and reported a greater number of abnormal reflexes in infants of both women who smoked and those who used e-cigarettes compared with those of women who did not smoke^56^. This could be related to differences between smoking and non-smoking mothers, or to tobacco exposure in early pregnancy, but could also be due to nicotine exposure.

Given the questions that remain about the potential risks of nicotine in pregnancy, stopping smoking without nicotine-containing aids is preferable to switching to such products. Only when the choice is between using nicotine products such as NRT or e-cigarettes or continuing to smoke, the use of NRT or e-cigarettes would be the recommended option.

As noted above, the much higher post-smoking-cessation nicotine use in the e-cigarette arm did not seem to affect birth outcomes. As in previous studies^30,57^, the women who used e-cigarettes were also reducing the nicotine content of their e-cigarettes over time. However, if e-cigarette use were to persist in the long term, it is likely to carry some health risks^58^, as well as maintaining nicotine dependence. In this scenario, e-cigarettes would represent a harm reduction approach. It is also not known whether, over an extended time period, e-cigarette use has positive or negative effects on the quality of life of ex-smokers and their rates of relapse. Longitudinal studies following up comparable cohorts of ex-smokers who do and do not use e-cigarettes are needed to provide this information.

In summary, in the unadjusted primary analysis there was insufficient evidence to confidently demonstrate that e-cigarettes are more effective than NRT in helping pregnant women to stop smoking. The effects of e-cigarettes appear to have been masked by e-cigarette use in the NRT arm. When abstinent participants using non-allocated products were excluded, e-cigarettes were markedly more effective than patches in all abstinence outcomes. The safety data provide some reassurance that for pregnant women who are unable to stop smoking unaided, e-cigarettes do not seem to pose more risk to birth outcomes assessed in this study than nicotine patches and may reduce the incidence of low birthweight.

## Methods

### Participants

The inclusion criteria included pregnancy (12–24 weeks of gestation), smoking daily, wanting help with stopping smoking, having no strong preference for NRT or e-cigarettes, and agreeing to use only the allocated stop-smoking product (and not the non-allocated product) for at least the first 4 weeks of their quit attempt.

Exclusion criteria included age <18 years old, allergy to nicotine skin patches, current daily use of NRT or e-cigarettes, and serious medical problems or high-risk pregnancy.

Recruitment was managed by research midwives in England and by the Stop Smoking Service in Scotland. Participants were identified from patient records and sent study information and invitation letters (alongside ultrasound scan appointment letters if appropriate) or invited via telephone, email or text; approached in person when attending antenatal hospital appointments; referred by community midwives or stop-smoking advisors; or self-referred via posters advertising the study at the sites’ antenatal clinics.

### Procedures

Potential participants were provided with study details that treated the two study arms in identical ways (Supplementary Data). Those interested in the trial were invited to the baseline visit. There, research midwives checked participants’ eligibility and collected informed consent. Participants then completed a baseline questionnaire, provided a saliva sample, and were randomized to one of the two study arms. The relevant product was shown and its use explained, and the date and time for the first support call was set up, typically in 1 week’s time. Participants were advised that the product would be posted to them in time for the first call. The site principal investigator then reviewed the participant’s documentation and confirmed their eligibility.

The study products were posted centrally from the Health and Lifestyle Research Unit (HAL). The HAL stop-smoking advisors and researchers also delivered up to six initial support calls (see the Behavioral Support section below) and collected the end of pregnancy and post-pregnancy follow-up data over the phone or via online or postal questionnaires. If the HAL staff could not reach participants, their smoking status at delivery was obtained from study sites where available. The study sites also reported on pregnancy outcomes.

The first follow-up was conducted towards the end of pregnancy. Given that the majority of pregnant women who smoke, abstain during pregnancy and return to smoking after delivery^46,59^, end of pregnancy follow-up calls were made at 35 weeks of gestation. Following an example from a recent trial^60^, to increase the chance of reaching participants the period for data collection was from 35 weeks of gestation to 10 weeks after the estimated delivery date. The effort put into collecting follow-up data was standardized (Supplementary Data).

At the end of pregnancy the participants reporting abstinence from smoking, concurrent use of cigarettes and e-cigarettes or NRT (dual use) or a reduction of cigarette consumption of 50% or more were asked to provide a saliva sample. Sampling kits were posted to them with a self-addressed envelope on the day their smoking status was established. Once the returned samples were received, participants were sent £20 for their time and effort. During the last 14 months of the study we also asked self-reported abstinent participants using nicotine-containing products to attend local study sites to provide a carbon monoxide reading.

An additional follow-up call was conducted at 3 months post-partum to establish smoking status at the end of pregnancy if this was not available from previous attempts at contact, and to collect self-reports of any new health problems or worsening of old health problems in the mother and infant. If any of these health problems met the definition of a serious adverse event, further information was retrieved from hospital records. Follow-up calls took place between April 2018 and September 2020.

The study was approved by the National Research Ethics Service Committee London – South East (ref: 17/LO/0962) and the Medicines and Healthcare Regulatory Agency via the CTIMP (Clinical Trial of an Investigational Medicinal Product) Notification Scheme. The trial was conducted in compliance with the Medicines for Human Use (Clinical Trials) Regulations 2004 (SI 2004/1031), Research Governance Framework, Good Clinical Practice guidelines, and the World Medical Association Declaration of Helsinki (1996). A Data Monitoring and Ethics Committee and a Trial Steering Committee supervised the study. The study was pre-registered (on 21 March 2017) in the International Standard Randomized Controlled Trial Number (ISRCTN) registry (ID no. ISRCTN62025374). The full protocol is at https://fundingawards.nihr.ac.uk/award/15/57/85 and a summary of the protocol amendments after study initiation is given in Supplementary Data.

### Study arms

#### E-cigarette

Participants were sent an EU Tobacco Product Directive-compliant refillable e-cigarette starter kit (One Kit by the UK E-cig Store), together with two 10 ml bottles of tobacco-flavored e-cigarette liquid (1.8% nicotine; 70% propylene glycol and 30% vegetable glycerol), a pack of five replacement coils, and an instruction leaflet (Supplementary Data). Further supplies of e-cigarette liquid were posted on request for up to 8 weeks. A lower strength e-cigarette liquid (1.1%) and e-cigarette liquid with fruit flavor were available as alternatives. Participants were encouraged to source e-cigarette liquids of the strength and flavor they liked, as well as different e-cigarette devices, and arrange their own supplies after 8 weeks if needed. The cost of the kit provided by the study was £22.75 and the cost of e-cigarette liquid was up to £24 for an 8 week supply.

#### Nicotine replacement treatment

Participants were sent an initial 2 week supply of Nicorette Invisi 15 mg 16 h nicotine patches with manufacturer instruction leaflets and instructed to apply patches every day upon waking, and remove them before bedtime. Further supplies were posted on request for up to 8 weeks. A lower strength patch (10 mg 16 h) was available as an alternative. Participants were encouraged to access further supplies themselves via their general practitioner or local Stop Smoking Service. This could be patches and/or other NRT products such as nicotine chewing gum, inhalator or mouth spray, to use in addition to the patch alone if needed. In the United Kingdom pregnant women who smoke receive NRT free of charge. The cost of patches provided by the study was up to £93.58 for an 8 week supply.

#### Behavioral support that accompanied both study arms

Participants received six phone calls from stop-smoking advisors who followed the practice of the UK Stop Smoking Service^61^. The first call explained their product use and helped to prepare them for their target quit date (TQD). The second call, conducted on or near the TQD, checked on any product issues and provided tips and strategies for smoking cessation. The further weekly calls checked on participants’ progress, product use and supplies, and offered guidance on maintaining abstinence or stopping smoking. The final call took place 4 weeks after the TQD. The first call took up to 20 min, the other calls took on average 10 min.

### Measures

At baseline, demographic details and smoking history were collected, including age in years, education (primary and secondary school only; further training but not university courses; higher (university) education), whether in paid employment, ethnicity (white British, white other, Asian Bangladeshi, Asian Indian, Asian Pakistani, Black African, Black Caribbean, mixed, other, do not wish to answer), number of cigarettes smoked per day, Fagerstrom test of cigarette dependence (FTCD, score range 0–10; ref. ^62^), whether living with another person who smokes, whether used NRT, varenicline and bupropion in the past, and whether tried e-cigarettes in the past. Participants also provided saliva samples for assessment of their cotinine levels.

At phone calls at weeks 1–4 after the TQD and at the end of pregnancy, participants reported on their smoking status and on allocated and non-allocated product use. At the end of pregnancy, saliva samples and carbon monoxide readings were collected as described above.

At each contact, including the call at 3 months post-partum, participants were asked about any health problems since the last call and reports were classified as serious adverse events, adverse events or adverse reactions. Sites were contacted when needed to check medical notes for clarification. Participants not using allocated products were asked for reasons and if these included physical symptoms, these were also recorded. Research midwives collected birth and maternal outcomes via hospital records and reported any birth-related serious adverse events and adverse events.

### Outcomes

The primary endpoint was prolonged abstinence from smoking from 2 weeks after the TQD until the end of pregnancy, defined as per Russell Standard^63^ (up to five lapses allowed with no smoking at all during the previous week at the time of final follow-up). This was validated using salivary cotinine (<10 ng ml^−1^) (ref. ^64^) for those not reporting using any nicotine product, or by salivary anabasine (<1 ng ml^−1^) (ref. ^65^) or carbon monoxide level <8 ppm for those reporting current use of e-cigarettes or NRT^66^. When there was a discrepancy between anabasine and carbon monoxide values, the carbon monoxide result was used. The Bedfont Pico carbon monoxide monitor was used at all study sites. Participants with missing validation as well as those lost to follow-up were included as non-abstinent participants.

Secondary endpoints included self-reported prolonged abstinence from smoking at the end of pregnancy, self-reported point prevalence abstinence (no smoking for at least the past 7 days) at 4 weeks and at the end of pregnancy, validated point prevalence abstinence at the end of pregnancy, and proportion of non-abstinent participants reducing their cigarette consumption by at least 50%. Participants who were reached only after delivery and who reported that they had now returned to smoking, but had been abstinent at delivery, were included as self-reported end of pregnancy abstinent participants, but as non-abstinent participants in the validated outcomes.

Regarding safety outcomes, we monitored serious adverse events, adverse events and adverse reactions and specifically the following: termination, miscarriage (non-live birth prior to 24 weeks of gestation), stillbirth (non-live birth at 24 weeks of gestation or later), neonatal death (from live birth to 28 days), post-neonatal death (from 29 days), preterm birth (<37 weeks of gestation), low birthweight (<2,500 g), neonatal intensive care admissions, congenital abnormalities, cesarean section delivery, birthweight and gestational age.

### Sample size

Using previous trials, the quit rate at delivery in the NRT arm was estimated at 8% (ref. ^13^) and that in the e-cigarette arm at 14% (ref. ^57^) (odds ratio = 1.87, RR = 1.75). To have 90% power (α = 0.05, two-tailed test) to detect this difference, 1,140 participants (570 in each condition) were needed.

### Randomization and blinding

An independent statistician developed the randomization sequence using permuted block randomization with a block size of at least 6 and a maximum of 12. The randomization list was accessible only to the independent statistician, on a secure server. Researchers conducting randomization used the database application to inform the participants of their study arm allocation. Researchers conducting follow-up calls were blind to treatment allocation until the follow-up contact was made. Once contact was made and the trial application was opened, condition-specific questions were visible on the computer screen. The trial statistician was blind to participant allocation until the analysis of the primary and secondary outcomes was complete. This was achieved by extracting and importing into Stata only the baseline characteristics, study arm and smoking status variables in the first stage of the analysis. Variables coding treatment adherence and product use were extracted only after the primary and secondary outcome analyses were completed.

### Statistical methods

The analysis plan was pre-registered on the Open Science Framework (https://osf.io/dvh4a). We report mean and standard deviation for continuous measures that are approximately symmetric, and median and quartiles if the distribution is skewed. Binary outcomes were analyzed using a binomial regression with a logarithmic link, which enables estimation of RR, calculated using the NRT arm as the reference. If the model were not to converge, we would use a Poisson regression model with robust standard errors. All tests were two-sided.

For the primary outcome we conducted three pre-specified sensitivity analyses: a per-protocol analysis that excluded participants who did not start product use or never established contact with the study team, an analysis in which we estimated missing data using multiple imputation by chained equation, and an analysis in which we excluded abstinent participants who used non-allocated products (that is, e-cigarettes in the NRT arm and NRT in the e-cigarette arm) for at least 5 consecutive days during the 4 weeks after the TQD or who reported end of pregnancy current use or regular use for at least 1 week or occasional use for at least 3 weeks.

The decision to exclude abstinent participants using non-allocated products rather than all such participants, the standard approach to account for contamination, was made to provide an estimate in both groups when the non-allocated treatment could not have contributed to abstinence, while avoiding bias. Given that different rates of product switching were expected in the two study arms (with a higher rate in the NRT arm), the exclusion of all participants who used non-allocated products would be likely to result in an overestimation of the cessation rate in the arm allocated to the less effective treatment and an underestimation of the difference between groups. To illustrate the effect of this, let us assume that the true quit rate is 10% with treatment A and 20% with treatment B and that the intervention is tested in a sample of 100 participants in each study arm. There will be 10 successful quitters in A and 20 in B. If all who fail with A (*n* = 90) try B and 20% succeed (*n* = 18) while half of those who fail with B try A (*n* = 40) and 10% succeed (*n* = 4), the quit rates will be 28% ((10 + 18)/100) and 24% ((20 + 4)/100) in the A and B arms, respectively, masking the real 10% versus 20% treatment difference. If we try to control for the bias by excluding all participants using non-allocated products (‘switchers’), this changes to even less accurate success rates of 100% (10/(100 − 90)) versus 33% (20/(100 − 40)). Exclusion of only abstinent switchers results in quit rates of 12% (10/(100 − 18)) versus 21% (20/(100 − 4)), the closest values to the true treatment effect.

In addition to the pre-specified sensitivity analysis that excluded abstinent participants using non-allocated products, we also conducted an exploratory sensitivity analysis that assumed that abstinent participants using non-allocated products would not succeed in stopping smoking without such use, and reclassified them as non-abstinent participants (Supplementary Table 8). This approach maintains randomization, enables the inclusion of the whole sample and maintains statistical power.

Regarding multiple imputation, its use in smoking cessation trials is problematic because missingness is not random^67^, but, given that it is often reported, we included it for completeness. Using multiple imputation by chained equation, we imputed missing data on self-reported sustained smoking status (that is, participant who smokes versus abstinent participant), biochemical validation results, and current nicotine use (yes versus no) to derive validated sustained abstinence (that is, the primary outcome). Imputation was done separately by randomized group and we generated 50 completed datasets^68,69^. The following auxiliary variables were included in the multiple imputation model, given that they were associated with either self-reported abstinence or the results of saliva assays or their missingness: FTCD, living with a person who smokes, number of cigarettes per day, education, occupation status, daily use of allocated products on all 4 intervention weeks, and point abstinence at week 4.

For the secondary outcomes, as for the primary outcome, sensitivity analyses were conducted excluding abstinent participants who used non-allocated products. We also conducted an exploratory analysis in which these participants were classified as non-abstinent participants (Supplementary Table 8).

Differences between the two study arms in adverse events, serious adverse events and adverse reactions, coded as present versus absent, were assessed using binomial regression with logarithmic link. The primary analysis was of singleton births. A sensitivity analysis that included multiple births estimated standard errors allowing for intragroup correlation and estimated 95% confidence intervals. To account for clustering at the mother level, a clustered sandwich estimator of the variance was applied.

When no adverse birth outcome was recorded, we assumed that none had occurred. For the safety analyses, the denominator excluded participants who withdrew from the study prior to delivery (*n* = 6).

We calculated the Bayes factor for key outcomes. This was not pre-specified, but was included to clarify whether the data support the null hypothesis or are insensitive. The Bayes factor indicates whether there is evidence for no effect or whether the data are insensitive in the case of a non-significant result^70^. We specified a half-normal distribution (that is, the top half of a normal distribution with mode = 0) with the standard deviation set to the expected effect size (that is, log relative risk). The expected effect size was based on our previous e-cigarettes versus NRT study for smoking cessation^57^ and on a study comparing nicotine and placebo patches for the effects of nicotine on birthweight^13^. For the primary outcome, the data were found to be insensitive (Bayes factor = 2.7). For the outcome excluding abstinent participants using non-allocated products and for the difference between the two study arms in the incidence of low birthweight, the effects were strong (Bayes factor = 10.0 and Bayes factor = 10.3, respectively).

### Reporting Summary

Further information on research design is available in the Nature Research Reporting Summary linked to this article.

## Online content

Any methods, additional references, Nature Research reporting summaries, source data, extended data, supplementary information, acknowledgements, peer review information; details of author contributions and competing interests; and statements of data and code availability are available at 10.1038/s41591-022-01808-0.

## Supplementary information

Supplementary Information Supplementary Tables 1–8

Reporting Summary

Supplementary Data Appendices 1–5

## Data Availability

Data requests from academic institutions should be submitted to the corresponding author, explaining the analyses planned. Anonymized data will be provided for additional analyses that do not overlap with analyses that the authors plan to conduct themselves.

## References

[1] Salihu HM, Wilson RE (2007). Epidemiology of prenatal smoking and perinatal outcomes. Early Hum. Dev..

[2] Rogers JM (2008). Tobacco and pregnancy: overview of exposures and effects. Birth Defects Res. C Embryo Today.

[3] Abraham M (2017). A systematic review of maternal smoking during pregnancy and fetal measurements with meta-analysis. PLoS One.

[4] Marufu TC, Ahankari A, Coleman T, Lewis S (2015). Maternal smoking and the risk of still birth: systematic review and meta-analysis. BMC Public Health.

[5] Yang I, Hall L (2019). Factors related to prenatal smoking among socioeconomically disadvantaged women. Women Health.

[6] Wisborg K, Henriksen TB, Jespersen LB, Secher NJ (2000). Nicotine patches for pregnant smokers: a randomized controlled study. Obstet. Gynecol..

[7] Pollak KI (2007). Nicotine replacement and behavioral therapy for smoking cessation in pregnancy. Am. J. Prev. Med..

[8] Oncken C (2008). Nicotine gum for pregnant smokers: a randomized controlled trial. Obstet. Gynecol..

[9] Oncken C (2019). Randomized trial of nicotine inhaler for pregnant smokers. Am. J. Obstet. Gynecol. MFM.

[10] Kapur B, Hackman R, Selby P, Klein J, Koren G (2001). Randomized, double-blind, placebo-controlled trial of nicotine replacement therapy in pregnancy. Curr. Therapeut. Res..

[11] Hotham ED, Gilbert AL, Atkinson ER (2006). A randomised-controlled pilot study using nicotine patches with pregnant women. Addict. Behav..

[12] El-Mohandes AA (2013). A randomized clinical trial of trans-dermal nicotine replacement in pregnant African-American smokers. Matern. Child Health J..

[13] Coleman T (2012). A randomized trial of nicotine-replacement therapy patches in pregnancy. N. Engl. J. Med..

[14] Berlin I, Grangé G, Jacob N, Tanguy M-L (2014). Nicotine patches in pregnant smokers: randomised, placebo controlled, multicentre trial of efficacy. BMJ.

[15] Stotts AL (2015). Randomized, controlled pilot trial of bupropion for pregnant smokers: challenges and future directions. Am. J. Perinatol..

[16] Nanovskaya TN (2017). Bupropion sustained release for pregnant smokers: a randomized, placebo-controlled trial. Am. J. Obstet. Gynecol..

[17] Claire R (2020). Pharmacological interventions for promoting smoking cessation during pregnancy. Cochrane Database Syst. Rev..

[18] Fish LJ (2009). Adherence to nicotine replacement therapy among pregnant smokers. Nicotine Tob. Res..

[19] Dempsey D, Jacob P, Benowitz NL (2002). Accelerated metabolism of nicotine and cotinine in pregnant smokers. J. Pharmacol. Exp. Ther..

[20] Bowker K, Lewis S, Coleman T, Cooper S (2015). Changes in the rate of nicotine metabolism across pregnancy: a longitudinal study. Addiction.

[21] Coleman T, Britton J, Thornton J (2004). Nicotine replacement therapy in pregnancy. BMJ.

[22] Hajek P, Etter JF, Benowitz N, Eissenberg T, McRobbie H (2014). Electronic cigarettes: review of use, content, safety, effects on smokers and potential for harm and benefit. Addiction.

[23] Saddleson M (2016). Enjoyment and other reasons for electronic cigarette use: results from college students in New York. Addict. Behav..

[24] Hajek P, Przulj D, Phillips A, Anderson R, McRobbie H (2017). Nicotine delivery to users from cigarettes and from different types of e-cigarettes. Psychopharmacology.

[25] Phillips-Waller A, Przulj D, Smith KM, Pesola F, Hajek P (2021). Nicotine delivery and user reactions to Juul EU (20 mg/ml) compared with Juul US (59 mg/ml), cigarettes and other e-cigarette products. Psychopharmacology.

[26] Hajek P, Przulj D, Phillips-Waller A, Anderson R, McRobbie H (2018). Initial ratings of different types of e-cigarettes and relationships between product appeal and nicotine delivery. Psychopharmacology.

[27] Carabello RS, Shafer PR, Patel D, Davis KC, McAfee TA (2017). Quit methods used by US adult cigarette smokers, 2014–2016. Prev. Chronic Dis..

[28] McNeill, A., Brose, L. S., Calder, R., Simonavicius, E. & Robson, D. *Vaping in England: An Evidence Update February 2021* (Public Health England, 2021).

[29] Hartmann-Boyce J (2021). Electronic cigarettes for smoking cessation. Cochrane Database Syst. Rev..

[30] Myers Smith K (2022). E-cigarettes versus nicotine replacement treatment as harm reduction interventions for smokers who find quitting difficult: randomized controlled trial. Addiction.

[31] Whittington JR (2018). The use of electronic cigarettes in pregnancy: a review of the literature. Obstet. Gynecol. Surv..

[32] Bowker K (2021). Pregnant women’s use of e‐cigarettes in the UK: a cross‐sectional survey. BJOG.

[33] Opondo C, Harrison S, Alderdice F, Carson C, Quigley MA (2021). Electronic cigarette use (vaping) and patterns of tobacco cigarette smoking in pregnancy: evidence from a population-based maternity survey in England. PLoS One.

[34] Dejmek J, Solanský I, Benes I, Lenícek J, Srám RJ (2000). The impact of polycyclic aromatic hydrocarbons and fine particles on pregnancy outcome. Environ. Health Perspect..

[35] Benowitz NL (2000). The use of pharmacotherapies for smoking cessation during pregnancy. Tob. Control.

[36] Benowitz NL, Dempsey DA (2004). Pharmacotherapy for smoking cessation during pregnancy. Nicotine Tob. Res..

[37] Bar‐Zeev Y, Lim LL, Bonevski B, Gruppetta M, Gould GS (2018). Nicotine replacement therapy for smoking cessation during pregnancy. Med. J. Aust..

[38] Fahy SJ, Cooper S, Coleman T, Naughton F, Bauld L (2014). Provision of smoking cessation support for pregnant women in England: results from an online survey of NHS Stop Smoking Services for pregnant women. BMC Health Serv. Res..

[39] Myers, K., McRobbie, H., West, O. & Hajek, P. *Smoking Cessation Interventions in Acute and Maternity Services: Review of Barriers and Facilitators* (National Institute for Health and Clinical Excellence, 2012).

[40] Taylor L (2021). Fetal safety of nicotine replacement therapy in pregnancy: systematic review and meta-analysis. Addiction.

[41] Eaton, D. L., Kwan, L. Y. & Stratton, K. (eds) *Public Health Consequences of E-Cigarettes* (National Academies Press, 2018). 29894118

[42] Koslow M, Petrache I (2020). A finale on EVALI?: The abated but not forgotten outbreak of acute respiratory illness in individuals who vape. JAMA Netw. Open.

[43] Bates, C. The outbreak of lung injuries often known as ‘EVALI’ was nothing to do with nicotine vaping. *Qeios*10.32388/ZGVHM7.3 (2021).

[44] Gartner C, Bonevski B, Hall W (2020). Miscommunication about the causes of the US outbreak of lung diseases in vapers by public health authorities and the media. Drug Alcohol Rev..

[45] Ussher M (2015). Physical activity for smoking cessation in pregnancy: randomised controlled trial. BMJ.

[46] Hajek P (2001). Randomized controlled trial of a midwife‐delivered brief smoking cessation intervention in pregnancy. Addiction.

[47] Berlin I, Singleton EG, Heishman SJ (2016). Craving and withdrawal symptoms during smoking cessation: comparison of pregnant and non-pregnant smokers. J. Subst. Abuse Treat..

[48] Lindson N (2019). Different doses, durations and modes of delivery of nicotine replacement therapy for smoking cessation. Cochrane Database Syst. Rev..

[49] Cooper S (2014). Effect of nicotine patches in pregnancy on infant and maternal outcomes at 2 years: follow-up from the randomised, double-blind, placebo-controlled SNAP trial. Lancet Respir. Med..

[50] Bruin JE, Gerstein HC, Holloway AC (2010). Long-term consequences of fetal and neonatal nicotine exposure: a critical review. Toxicol. Sci..

[51] Rowell PP, Clark MJ (1982). The effect of chronic oral nicotine administration on fetal weight and placental amino acid accumulation in mice. Toxicol. Appl. Pharmacol..

[52] Cohen G (2005). Perinatal exposure to nicotine causes deficits associated with a loss of nicotinic receptor function. Proc. Natl Acad. Sci. USA.

[53] Dempsey DA, Benowitz NL (2001). Risks and benefits of nicotine to aid smoking cessation in pregnancy. Drug Saf..

[54] Regan AK, Pereira G (2021). Patterns of combustible and electronic cigarette use during pregnancy and associated pregnancy outcomes. Sci. Rep..

[55] McDonnell B, Dicker P, Regan C (2020). Electronic cigarettes and obstetric outcomes: a prospective observational study. BJOG.

[56] Froggatt S, Reissland N, Covey J (2020). The effects of prenatal cigarette and e-cigarette exposure on infant neurobehaviour: a comparison to a control group. EClinicalMedicine.

[57] Hajek P (2019). A randomized trial of e-cigarettes versus nicotine-replacement therapy. N. Engl. J. Med..

[58] McNeill, A., Brose, L., Calder, R., Bauld, L. & Robson, D. *Vaping in England: An Evidence Update Including Mental Health and Pregnancy* (Public Health England, 2020).

[59] Jones M, Lewis S, Parrott S, Wormall S, Coleman T (2016). Re‐starting smoking in the postpartum period after receiving a smoking cessation intervention: a systematic review. Addiction.

[60] Whitemore R (2019). Effectiveness and cost-effectiveness of a tailored text-message programme (MiQuit) for smoking cessation in pregnancy: study protocol for a randomised controlled trial (RCT) and meta-analysis. Trials.

[61] McEwen, A., Hajek, P., McRobbie, H. & West, R. *Manual of Smoking Cessation: A Guide for Counsellors and Practitioners* (Blackwell Publishing, 2006).

[62] Fagerström K (2011). Determinants of tobacco use and renaming the FTND to the Fagerström Test for Cigarette Dependence. Nicotine Tob. Res..

[63] West R, Hajek P, Stead L, Stapleton J (2005). Outcome criteria in smoking cessation trials: proposal for a common standard. Addiction.

[64] SRNT Subcommittee on Biochemical Verification (2002). Biochemical verification of tobacco use and cessation. Nicotine Tob. Res..

[65] Brown J (2014). Internet-based intervention for smoking cessation (StopAdvisor) in people with low and high socioeconomic status: a randomised controlled trial. Lancet Respir. Med..

[66] Benowitz NL (2020). Biochemical verification of tobacco use and abstinence: 2019 update. Nicotine Tob. Res..

[67] Hajek P, West R (2010). Commentary on Smolkowski et al. (2010): why is it important to assume that non‐responders in tobacco cessation trials have relapsed?. Addiction.

[68] Sullivan TR, White IR, Salter AB, Ryan P, Lee KJ (2018). Should multiple imputation be the method of choice for handling missing data in randomized trials?. Stat. Methods Med. Res..

[69] White IR, Royston P, Wood AM (2011). Multiple imputation using chained equations: issues and guidance for practice. Stat. Med..

[70] Beard E, Dienes Z, Muirhead C, West R (2016). Using Bayes factors for testing hypotheses about intervention effectiveness in addictions research. Addiction.

