# Author Correction: Electronic cigarettes versus nicotine patches for smoking cessation in pregnancy: a randomized controlled trial

**DOI:** 10.1038/s41591-022-02099-1

**Published:** 2022-11-07

**Authors:** Peter Hajek, Dunja Przulj, Francesca Pesola, Chris Griffiths, Robert Walton, Hayden McRobbie, Tim Coleman, Sarah Lewis, Rachel Whitemore, Miranda Clark, Michael Ussher, Lesley Sinclair, Emily Seager, Sue Cooper, Linda Bauld, Felix Naughton, Peter Sasieni, Isaac Manyonda, Katie Myers Smith

**Affiliations:** 1 Wolfson Institute of Population Health, Queen Mary University of London, London, UK; 2 National Drug and Alcohol Research Centre, University of New South Wales, Sydney, New South Wales Australia; 3 Faculty of Medicine and Health Sciences, University of Nottingham, Nottingham, UK; 4 Division of Population Heath Sciences and Education, St Georges, University of London, London, UK; 5 Institute of Social Marketing and Health, University of Stirling, Stirling, UK; 6 Usher Institute and SPECTRUM Consortium, University of Edinburgh, Edinburgh, UK; 7 School of Health Sciences, University of East Anglia, Norwich, UK; 8 The Cancer Research UK and King’s College London Cancer Prevention Trials Unit, King’s College London, London, UK; 9 St George’s Healthcare NHS Trust, London, UK

**Keywords:** Medical research, Diseases

Correction to: *Nature Medicine* 10.1038/s41591-022-01808-0, published online 16 May 2022.

In the version of this article initially published, there was an incorrect statistic in the “Secondary outcomes” section of the Results: “63 of 171” should have read “63 of 571.” In the “Study arms” section of the Methods, “18%” should have read “1.8%” and “11%” should have read “1.1%.”

The affiliation for Peter Sasieni was incorrectly listed as “Kings Clinical Trials Unit, Kings College London, London, UK” instead of “The Cancer Research UK and King’s College London Cancer Prevention Trials Unit, King’s College London, London, UK.”

The acknowledgements were missing the sentence “For part of the trial, F.P. was supported by Cancer Research UK (grant no. C8162/A25356).”

The changes have been made to the HTML and PDF versions of the article.

