# Supplementary material for: Electronic cigarettes versus nicotine patches for smoking cessation in pregnancy: a randomized controlled trial
Source: Nat Med. 2022 May 16;28(5):958–64. doi: 10.1038/s41591-022-01808-0 (PMC9117131; doi:10.1038/s41591-022-01808-0)
Supplement: Supplementary file 1 — Supplementary Tables 1–8 [file 41591_2022_1808_MOESM1_ESM.pdf]

nature portfolio

https://doi.org/10.1038/s41591-022-01808-0

Supplementary information

Electronic cigarettes versus nicotine patches for smoking cessation in pregnancy: a randomized controlled trial

In the format provided by the authors and unedited

Supplementary tables

Supplementary Table 1: Use of non-allocated products in the two study arms

	**E-cigarettes (571)**	**NRT (569)**
Use of non-allocated product, N (%)	16 (2.8%)	101 (17.8%)
Users of non-allocated product among validated abstainers at EOP, N (% of abstainers)	0 (0%)	5 (20.0%)
Users of non-allocated product among self-reported sustained abstainers at EOP, N (% of abstainers)	2 (3.2%)	13 (29.5%)
Users of non-allocated product among self-reported 7-day abstainers at EOP, N (% of abstainers)	6 (5.1%)	25 (32.1%)

EOP=end of pregnancy

Supplementary Table 2. Smoking reduction in non-abstainers

	**E-cigarettes (N=453)**	**NRT (N=491)**	**RR (95%CIs)[#]**
Validated* 50% reduction at EOP	12 (2.7)	12 (2.4)	1.08 (0.49-2.39) p=0.84
Sensitivity analysis			
Reducers using non-allocated product excluded (N=453 and N=489)	12 (2.7)	10 (2.0)	1.30 (0.57-2.97) p=0.54
Self-reported 50% reduction at EoP	192 (42.4)	166 (33.8)	**1.25 (1.06- 1.48) p=0.007**
Sensitivity analysis			
Reducers using non-allocated product excluded (N=448 and N=450)	187 (41.7)	125 (27.8)	**1.50 (1.25-1.81) p<0.001**

* 50+% reduction in cotinine levels compared to baseline.
[#] Relative risks obtained using a binomial regression with a logarithmic link with two-sided p-values
EOP=end of pregnancy

Supplementary Table 3. E-cigarettes use in the e-cigarettes arm

Products used during the initial 4 weeks (n=344) [#] ******* N (%)	Refillable e-cigarettes	324 (94.2)
	Cig-a-like	1 (0.3)
	Cartridge/Pod	1 (0.3)
	Information missing	18 (5.2)
Nicotine strength N (%)	0 mg/mL	7 (2.0)
	1-10mg/mL	47 (13.7)
	11-20mg/mL	199 (57.9)
	Information missing	91 (26.5)
Flavour N (%)	Fruit	180 (52.3)
	Tobacco	24 (7.0)
	Mint/menthol	22 (6.4)
	Chocolate, dessert, candy	11 (3.2)
	Other	21 (6.1)
	Information missing	86 (25.0)
Products used since last contact at EOP (N=371) N (%)	Refillable e-cigarettes	330 (89.0)
	Cig-a-like	0 (0)
	Cartridge/Pod	2 (0.5)
	Information missing	39 (10.5)
Nicotine strength N (%)	0 mg/ml	8 (2.2)
	1-10mg/ml	77 (20.8)
	11-20mg/ml	61 (16.4)
	Information missing	225 (60.7)
Flavour N (%)	Fruit	97 (26.2)
	Mint/menthol	38 (10.2)
	Chocolate, dessert, candy	19 (5.1)
	Tobacco	17 (4.6)
	Other	24 (6.5)
	Information missing	176 (47.4)

[#]Participants were coded as having used e-cigarettes if they reported any use during at least one of the weeks; while at end of pregnancy if they reported having used their allocated product since the last call.

* If several products were used, only the last product used is listed

EOP=end of pregnancy

Supplementary Table 4: Birth outcomes by study arms, including twin births (9 in e-cigarette arm and 4 in NRT arm)

	E-cigarettes (N=564)^	NRT (N=557)^	RR (95% CI)**
Miscarriage N (%)	3 (0.5)	3 (0.5)	0.99 (0.20-4.87) p=0.99
Stillbirth N (%)	2 (0.4)	0 (0)	N/C$
Neonatal death N (%)	2 (0.4)	3 (0.5)	0.66 (0.11–3.93) p=0.65
Post-neonatal death N (%)	0	3 (0.5)	N/C$
Maternal death N (%)	0	0	N/C$
Preterm birth N (%)	56 (9.9)	69 (12.4)	0.80 (0.56-1.14) p=0.22
Low birthweight N (%)<br>N: 558 vs. 549	63 (11.3)	86 (15.7)	**0.72 (0.53-0.99) p=0.04**
NICU admission N (%)	58 (10.3)	46 (8.3)	1.25 (0.85-1.81) p=0.25
Congenital abnormalities N (%) #	26 (4.6)	15 (2.7)	1.71 (0.92-3.20) p=0.09
Terminations N (%)<br>-Due to congenital abnormalities<br><br>-Due to premature rupture of membranes	 1 (0.2)<br><br>2 (0.4)	 2 (0.4)<br><br>0	 1.48 (0.25–8.84) p=0.67<br>N/C$
Total number of adverse birth outcomes	213	227	
Number of women with adverse birth outcomes N (%)<br>(N=555 vs 553)	120 (21.6)	122 (22.1)	0.98 (0.78-1.22) p=0.86
Delivery by cesarean section N (%)	145 (25.7)	152 (27.2)	0.94 (0.77-1.15) p=0.56
Gestational age – weeks Mean (SD) N: 562 vs. 555	38.3 (3.1)	38.2 (3.1)	0.12 (-0.25-0.49)* p=0.52
Birthweight in kg Mean (SD)<br>N: 558 vs. 549	(0.63)	3.1 (0.63)	0.01 (-0.07-0.08)* p=0.86

^ Number of babies
# 2 infants in the e-cigarettes arm and 1 in the NRT arm had 2 congenital abnormalities
$ Not calculated
* Mean difference (95%CIs)
** Relative risks obtained using a binomial regression with a logarithmic link with two-sided p-values

Supplementary Table 5: Other serious adverse events (SAEs), adverse events (AEs) and adverse reactions (ARs)

Event	E-cigarettes	NRT
Other SAEs mother		
Premature rupture of the membranes	5	5
Pre-eclamspia	3	3
Threatened labour	3	3
Vaginal haemorrhage	2	4
Genitourinary tract infection	0	4
Haemorrhage in pregnancy	2	2
Abdominal pain	2	1
Migraine	1	2
Premature labour	1	2
Other (see list)	18	21
Other SAEs baby		
Newborn Respiratory Disorders	9	7
Jaundice	3	2
Vomiting	2	2
Meconium aspiration syndrome	3	1
Drug withdrawal syndrome	3	1
Sepsis neonatal	4	0
Hypoglycemia neonatal	3	0
Tonsillitis	2	1
Foetal growth restriction	1	2
Other (see list)	20	20
AEs mother		
Nasopharyngitis	25	17
Lower respiratory tract infection	15	9
Nausea	12	11
Headache	11	9
Cough	8	8
Gestational diabetes	6	11
Influenza like illness	7	6
Migraine	2	7
Urinary tract infection	3	5
Abortion induced	4	2
Perinatal depression	4	2
Vaginal hemorrhage	4	2
Asthma	2	3
Oropharyngeal pain	1	4
Vomiting	3	2
Hypertension	0	4
Viral infection	2	2
Abdominal pain upper	3	1
Depression	1	2

Dyspepsia<br>Hypotension<br>Other	1<br>1<br>48	2<br>2<br>35
AEs baby		
Foetal Growth Restrictions<br>Other	1<br>4	2<br>8
Total number of other mother/infant SAEs and AEs<br>N (%)	255	239
Number of participants with other SAEs and AEs N (%)	181	162
ARs potentially related to treatment		
Application site irritation, hypoesthesia ,rash, pain, or<br>pruritus<br>Nausea<br>Cough<br>Oropharyngeal pain or irritation<br>Rash<br>Headache<br>Dizziness<br>Chest pain or discomfort<br>Vomiting<br>Dyspnea<br>Migraine<br>Myalgia<br>Other (see list)	0<br>17<br>42<br>39<br>0<br>4<br>1<br>11<br>1<br>3<br>1<br>0<br>7	81<br>36<br>0<br>0<br>14<br>9<br>8<br>0<br>3<br>0<br>2<br>3<br>6
Total number of ARs	126	162
Number of participants with ARs *	108	148
*Action following ARs***		
Study drug discontinuation/interruption following AR	36	111
Study drug dose change following AR	41	12

* RR=0.86, 95%CI: 0.74-1.01

** $Chi^2_{(1)}$=46.0, p<.001 (two-sided p-value)

Note: Cases of conditions that led to hospitalization are listed under SAEs while those that did not are considered AEs. The same condition (e.g. foetal growth restriction) may thus appear under different headings.

Supplementary Table 6: SAE and AE that occurred only once or twice

Event	E-cigarettes	NRT
**Other SAEs mother**		
Abdominal pain upper	1	0
Acute myocardial infarction	1	0
Alcoholism	1	0
Cellulitis	0	1
Cerebral haemorrhage	1	0
Cervix inflammation	0	1
Dehydration	1	0
Diarrhoea	0	1
Eclampsia	0	1
Endometritis decidual	0	1
Epilepsy	1	1
Gastritis	0	1
Gestational diabetes	0	1
Haemoglobin decreased	0	1
Hyperemesis gravidarum	0	1
Influenza	1	1
Kidney infection	1	0
Lower respiratory tract infection	0	1
Mastitis	1	0
Nephrolithiasis	1	0
Pneumonia	1	1
Postpartum haemorrhage	1	0
Premature separation of placenta	0	1
Preterm premature rupture of membranes	0	1
Puerperal pyrexia	1	0
Pulmonary thrombosis	0	1
Pyelonephritis acute	0	1
Renal pain	1	0
Retained products of conception	0	1
Sciatica	1	0
Sepsis	1	1
Tooth infection	0	1
Upper respiratory tract infection	0	1
Ureteric injury	1	0
Wound infection	1	0
Other SAEs baby		
Abdominal distension	1	0
Asthma	1	0
Benign Neonatal Sleep Myoclonus	0	1
Beta haemolytic streptococcal infection	1	0
Bradycardia	1	0
Bronchiolitis	0	1

Bronchitis	0	1
Cardiac arrest neonatal	0	1
Cholecystectomy	0	1
Foetal cardiac arrest	1	0
Foetal hypokinesia (1)	1	1
Haematoma	0	1
Hospitalisation for further diagnosis	1	0
Hypertonia neonatal	0	1
Hypothermia neonatal	1	1
Hypoxic-ischaemic encephalopathy	1	0
Immune thrombocytopenia	1	0
Infantile apnoea	1	1
Intraventricular haemorrhage neonatal	0	1
Low birthweight baby	1	1
Necrotising enterocolitis neonatal	1	0
Neonatal infection	1	1
Neonatal pneumothorax	0	1
Neonatal seizure	2	0
Perinatal stroke	0	1
Poor feeding infant	0	1
Poor weight gain neonatal	0	1
Shoulder dystocia	0	1
Skin discolouration	1	0
Skull fracture	0	1
Spinal cord neoplasm	1	0
Viral infection	2	0
**AEs mother**		
Abdominal pain	0	2
Abdominal pain lower	0	1
Anaemia of pregnancy	2	0
Application site irritation	2	0
Abdominal discomfort	1	0
Acne	1	0
Acute sinusitis	1	0
Anxiety	1	1
Back pain	1	1
Bartholin's abscess	1	0
Bile output increased	0	1
Bronchitis	0	1
Chest discomfort	1	1
Chest pain	1	0
Cholestasis	1	1
Constipation	1	1
Crohn's disease	0	1
Deep vein thrombosis	1	0
Decreased appetite	0	1

Depressed mood	0	1
Dermatitis allergic	1	0
Diarrhoea	1	0
Dizziness	1	1
Dyspnoea	1	1
Ear infection	1	0
Eczema	1	0
Fatigue	1	0
Fluid retention	1	0
Food poisoning	1	0
Gastroenteritis	0	1
Haemorrhage urinary tract	0	1
Iron deficiency	1	0
Kidney infection	1	0
Ligament injury	1	1
Mastitis	1	0
Mental disorder	0	1
Mood swings	0	1
Mouth ulceration	1	0
Muscle spasms	0	1
Neck pain	1	0
Oedema peripheral	1	0
Oligohydramnios	1	0
Otitis media acute	1	0
Pain	0	1
Palpitations	0	1
Panic attack	1	1
Pelvic pain	1	0
Peripheral swelling	0	1
Placenta praevia	1	0
Post procedural complication	0	1
Pre-eclampsia	1	0
Premature rupture of membranes	0	1
Pulmonary embolism	1	0
Pyrexia	1	0
Rash	0	1
Rhesus antibodies	0	1
Road traffic accident	1	0
Seasonal allergy	1	0
Sinusitis	0	1
Suicidal ideation	0	1
Subcutaneous abscess	1	0
Symphysiolysis	0	1
Thrombosis	1	0
Tonsillitis	0	1
Tooth extraction	0	1
Tooth fracture	1	0
Wheezing	1	0
**AEs baby**		
Acid Reflux	1	0

Bilateral ventriculomegaly	0	1
Bronchitis	1	1
Chesty Cough	0	1
Cyst, NOS	0	1
Difficulty breathing	0	1
Foetal hypokinesia	1	0
Infection	0	1
Respiratory distress	0	1
Viral infection	1	1
**ARs potentially related to treatment**		
Arthralgia	0	1
Asthma	2	0
Dyspepsia	2	0
Eczema	0	1
Functional gastrointestinal disorder	0	1
Hyperhidrosis	0	1
Lip swelling	1	0
Muscle swelling	0	1
Nightmare	0	1
Stomatitis	1	0
Wheezing	1	0

Supplementary Table 7. Median time to follow-up in the two study arms

	E-cigarettes<br>N=505	NRT<br>N=490	Wilcoxon rank sum#
Follow-up completed pre-delivery	-23 days (IQR=-31 to -14; N=318)	-23 days (IQR=-31 to -16; N=282)	z=-0.4, p=0.68.
Follow-up completed post-delivery	16 days (IQR=0 to +66; N=187)	10 days (IQR= 0 to +36; N=208)	z=2.0, p=0.04

Two-sided p-value
Note 1: 17 participants had no date of delivery recorded and 2 had the date of follow-up missing
Note 2: There were 395 women who answered the follow-up calls only after delivery, 187 (37%) of the EC arm vs 208 (42%) of the NRT arm (chi(1)=3.1, p=0.08). Of these women, 12 (6.4%) vs 9 (4.3%) were self-reported abstainers (chi(1)=0.85, p=0.36). Among these, 5 (41.7%) vs 3 (33.3%) reported relapsing back to smoking since delivery (chi(1)=0.15, p=0.70).

Supplementary Table 8: Sensitivity analyses of abstinence outcomes counting abstinence only if not accompanied by regular use of non-allocated product

	**E-cigarettes (N=571)**	**NRT (N=569)**	**RR (95%CIs)#**
Validated prolonged abstinence at EoP	39 (6.8)	20 (3.5)	1.94 (1.15- 3.29)
Self-reported prolonged abstinence at EoP	61 (10.7)	31 (5.5)	1.96 (1.29-2.97)
Validated point-prevalence abstinence at EoP	56 (9.8)	29 (5.1)	1.92 (1.25-2.97)
Self-reported point-prevalence abstinence at EoP	112 (19.6)	53 (9.3)	2.11 (1.55-2.86)
Self-reported abstinence at 4 weeks	88 (15.4)	48 (8.4)	1.83 (1.31-2.55)

# Relative risks obtained using a binomial regression with a logarithmic link with two-sided p-values.
EOP=end of pregnancy
