# Supplementary material for: Electronic cigarettes versus nicotine patches for smoking cessation in pregnancy: a randomized controlled trial
Source: Nat Med. 2022 May 16;28(5):958–64. doi: 10.1038/s41591-022-01808-0 (PMC9117131; doi:10.1038/s41591-022-01808-0)
Supplement: Supplementary file 2 — Reporting Summary [file 41591_2022_1808_MOESM2_ESM.pdf]

nature research

Corresponding author(s): Francesca Pesola

Last updated by author(s): Mar 30, 2022

Reporting Summary

Nature Research wishes to improve the reproducibility of the work that we publish. This form provides structure for consistency and transparency in reporting. For further information on Nature Research policies, see our Editorial Policies and the Editorial Policy Checklist.

Statistics

For all statistical analyses, confirm that the following items are present in the figure legend, table legend, main text, or Methods section.

n/a	Confirmed	
☐	☒	The exact sample size (*n*) for each experimental group/condition, given as a discrete number and unit of measurement
☐	☒	A statement on whether measurements were taken from distinct samples or whether the same sample was measured repeatedly
☐	☒	The statistical test(s) used AND whether they are one- or two-sided<br>*Only common tests should be described solely by name; describe more complex techniques in the Methods section.*
☐	☒	A description of all covariates tested
☐	☒	A description of any assumptions or corrections, such as tests of normality and adjustment for multiple comparisons
☐	☒	A full description of the statistical parameters including central tendency (e.g. means) or other basic estimates (e.g. regression coefficient) AND variation (e.g. standard deviation) or associated estimates of uncertainty (e.g. confidence intervals)
☐	☒	For null hypothesis testing, the test statistic (e.g. *F*, *t*, *r*) with confidence intervals, effect sizes, degrees of freedom and *P* value noted<br>*Give P values as exact values whenever suitable.*
☒	☐	For Bayesian analysis, information on the choice of priors and Markov chain Monte Carlo settings
☒	☐	For hierarchical and complex designs, identification of the appropriate level for tests and full reporting of outcomes
☒	☐	Estimates of effect sizes (e.g. Cohen's *d*, Pearson's *r*), indicating how they were calculated

Our web collection on statistics for biologists contains articles on many of the points above.

Software and code

Policy information about availability of computer code

Data collection	Data were collected using a custom-made web-based database application created by the Barts Clinical Trials Unit
Data analysis	Data were analysed using Stata 16.1

For manuscripts utilizing custom algorithms or software that are central to the research but not yet described in published literature, software must be made available to editors and reviewers. We strongly encourage code deposition in a community repository (e.g. GitHub). See the Nature Research guidelines for submitting code & software for further information.

Data

Policy information about availability of data

All manuscripts must include a data availability statement. This statement should provide the following information, where applicable:

- Accession codes, unique identifiers, or web links for publicly available datasets
- A list of figures that have associated raw data
- A description of any restrictions on data availability

Data requests from academic institutions should be submitted to the corresponding author, explaining the analyses planned. Anonymised data will be provided for additional analyses that do not overlap with analyses the authors plan to conduct themselves.

Field-specific reporting

Please select the one below that is the best fit for your research. If you are not sure, read the appropriate sections before making your selection.

☐ Life sciences ☒ Behavioural & social sciences ☐ Ecological, evolutionary & environmental sciences

For a reference copy of the document with all sections, see nature.com/documents/nr-reporting-summary-flat.pdf

Behavioural & social sciences study design

All studies must disclose on these points even when the disclosure is negative.

Study description	Randomised controlled trial; quantitative data.
Research sample	Pregnant smokers seeking help to quit smoking, aged 18 years and over; representative sample. The trial aimed to test two interventions to help pregnant smokers to quit smoking.
Sampling strategy	Convenient sample. We estimated from previous trials that quit rate at delivery would be 81% in the NRT arm and 14% in the EC arm (odds ratio 1.87; RR = 1.75). To have 90% power (alpha=0.05; two-tailed), 1,1140 participants (570 in each condition) would be needed to detect this difference,
Data collection	At baseline data were collected by researchers, not blind to study allocation. Also to note that the site PI, who made the medical assessment on eligibility after randomisation, was blind to allocation to prevent bias. Researchers conducting follow-up calls were blind to treatment allocation until the follow-up contact was made. Once contact was made and the trial application was opened, condition-specific questions were visible on the computer screen. Some follow-up questionnaires were self-completed by the participants.
Timing	January 2018 to September 2020.
Data exclusions	No exclusions for primary and secondary smoking abstinence measures; however, 2 participants with elective termination were excluded from the analysis of safety (birth and maternal outcome analyses) as described in the methods.
Non-participation	Six women withdrew consent before end of pregnancy so their data could not be used in the analyses of birth and maternal outcomes. They are assumed to be smoking at end of pregnancy in the primary and secondary analysis of smoking abstinence.<br><br>Reasons for withdrawing consent:<br><br>3 participants did not specify a reason (decision is unknown; all 3 in patch arm)<br><br>1 participant said the product was not helping (patch arm)<br><br>1 participant said she did not want to use patches (patch arm)<br><br>1 participants reported she did not want to take part anymore (e-cigarettes)
Randomization	A sequence of blocks of random size with equal numbers assigned to each arm within each block was programmed into the study database application. The randomisation allocation sequence was generated by the study statistician. Researchers conducting randomisation, informed participants of the study arm they had been allocated to by the database application.

Reporting for specific materials, systems and methods

We require information from authors about some types of materials, experimental systems and methods used in many studies. Here, indicate whether each material, system or method listed is relevant to your study. If you are not sure if a list item applies to your research, read the appropriate section before selecting a response.

Materials & experimental systems

n/a	Involved in the study	
☒	☐	Antibodies
☒	☐	Eukaryotic cell lines
☒	☐	Palaeontology and archaeology
☒	☐	Animals and other organisms
☐	☒	Human research participants
☐	☒	Clinical data
☒	☐	Dual use research of concern

Methods

n/a	Involved in the study	
☒	☐	ChIP-seq
☒	☐	Flow cytometry
☒	☐	MRI-based neuroimaging

Human research participants

Policy information about studies involving human research participants

Population characteristics	See above
Recruitment	Participants were recruited from 23 hospital sites across England, and one NHS Stop Smoking Service (SSS) in Scotland. Recruitment was managed by research midwives in England and by the SSS in Scotland. Participants were identified from patient records and sent study information and invitation letters (alongside ultrasound scan appointment letters if appropriate) or invited via telephone, email or text; approached in person when attending antenatal hospital appointments; referred by community midwives or stop-smoking advisors; or self-referred via posters advertising the study at the sites' antenatal clinics.<br><br>Participants may have had different expectations regarding the two study products, we tried to mitigate this potential bias by only including participants who were willing to use either product and by avoiding any indication that one product may be superior to the other in information to participants, but more participants in the NRT arm never started product use. Engagement with treatment could also affect the response to follow-up calls. More participants in the NRT arm answered the follow-up calls only after delivery, though the difference did not reach statistical significance and the time lapse between delivery and follow-up was shorter in the NRT arm.
Ethics oversight	The study was approved by the National Research Ethics Service Committee London –South East (ref: 17/LO/0962) and the MHRA via the CTIMP Notification Scheme. The trial was conducted in compliance with the Medicines for Human Use (Clinical Trials) Regulations 2004 (SI 2004/1031), Research Governance Framework, GCP guidelines, and the World Medical Association Declaration of Helsinki (1996).

Note that full information on the approval of the study protocol must also be provided in the manuscript.

Clinical data

Policy information about clinical studies

All manuscripts should comply with the ICMJE guidelines for publication of clinical research and a completed CONSORT checklist must be included with all submissions.

Clinical trial registration	ISRCTN62025374
Study protocol	https://fundingawards.nihr.ac.uk/award/15/57/85
Data collection	Baseline data were collected in person in antenatal clinics. The remaining data, including follow up data, were collected over the phone or via online or postal questionnaires. Birth and maternal outcomes were collected from hospital medical records. Recruitment began in January 2018 and ended in November 2019. Follow-up took place between April 2018 and September 2020.
Outcomes	The primary endpoint was prolonged abstinence from smoking from 2 weeks after TQD (target quit date) until EOP (end of pregnancy), defined as per Russel Standard, validated by salivary cotinine l(<10 ng/ml) for those not reporting using any nicotine product, or by salivary anabasine (<1 ng/m) or CO levels for those reporting current use of e-cigarettes or NRT. Participants with missing validation as well as those lost to follow-up were included as non-abstainers.<br><br>Secondary endpoints included self-reported prolonged abstinence from smoking at EOP, self-reported point prevalence abstinence (no smoking for at least the past 7 days) at 4 weeks and EOP, validated point prevalence abstinence at EOP, and proportion of non-abstinent participants reducing their cigarette consumption by at least 50%.<br><br>To be classified as 50% reducers, smokers had to report at least a 50% reduction in the number of cigarettes smoked at EOP compared to baseline. Participants with missing information were coded as not having achieved 50% reduction.<br><br>Self-reported prolonged abstinence at EOP was defined as a report of having smoked no more than 5 cigarettes at the relevant endpoint, with no smoking at all over the past 7 days but biochemical validation was not required. Participants with missing information on the endpoint were coded as not abstinent.<br><br>Regarding safety outcomes, we monitored serious adverse events (SAEs), adverse events (AEs), and adverse reactions (ARs) and specifically the following: termination, miscarriage (non-live birth prior to 24 weeks gestation), stillbirths (non-live birth at 24 weeks gestation or later), neonatal death (from live birth to 28 days), post-neonatal death (from 29 days), preterm birth (<37 weeks gestation), low birthweight (<1,500g), neonatal intensive care admissions (NICU), congenital abnormalities, caeserian-section delivery, birthweight, and gestational age.
