# Supplementary material for: Electronic cigarettes versus nicotine patches for smoking cessation in pregnancy: a randomized controlled trial
Source: Nat Med. 2022 May 16;28(5):958–64. doi: 10.1038/s41591-022-01808-0 (PMC9117131; doi:10.1038/s41591-022-01808-0)
Supplement: Supplementary file 3 — Appendices 1–5 [file 41591_2022_1808_MOESM3_ESM.pdf]

Appendix 1: Patient Information Sheet

Queen Mary
University of London

INVITATION TO PARTICIPATE IN A RESEARCH PROJECT

Helping Pregnant Smokers Quit: A Multi-Centre RCT of Electronic Cigarette and Nicotine Patches

Barts and The London School of Medicine and Dentistry,
Queen Mary, University of London

(REC ref: 17/LO/0962; IRAS ID:220190)

We would like to invite you to take part in a research study. The information which follows tells you about it. It is important that you understand what is in this leaflet. Please ask any questions you want to about the research and we will try our best to answer them.

The Study

In this study we want to find out whether weekly phone calls, together with an e-cigarette (EC) *or* a nicotine patch are effective in helping pregnant women stop smoking. If you decide to take part in the study, a computer will decide at random (by chance) which of these treatments you will receive.

What are E-cigarettes and nicotine patches?

Nicotine patches are placed on the body and provide nicotine throughout the day so smokers don't feel the urge to smoke as much. Patches are put on in the morning and taken off before going to bed. E-cigarettes (EC) are battery-operated devices that provide nicotine in a vapour that looks like smoke. They can be used throughout the day to reduce the need to smoke. Both products are considered safe when used temporarily to help with stopping smoking.

What will happen if you take part?

You will be asked to attend one session with research staff which can happen at the same time as your ultrasound or other routine appointments. After this, your first supply of patches or EC will be posted to you and a stop smoking advisor will call you on a weekly basis for 6 weeks to check on your progress and provide advice and support.

The study will last between 7 to 10 months (including the final follow-up) depending on what stage in your pregnancy you join the study. The table below provides details of what will happen throughout the study.

Session 1	You will ***see the research midwife/nurse/stop smoking advisor*** and they will describe the study and answer any questions. Your consent to take part in the study and information about your smoking will be collected. They will also take a saliva sample to measure the amount of nicotine you are getting from your cigarettes.<br><br>You will then be allocated to one of the two treatment groups: EC or Patches. This is decided at random by a computer.<br><br>The research staff will explain how to use your allocated product and will set a date and time for the stop smoking advisor to call you.<br><br>A two-week supply of your study product (EC/patch) will be posted to you in the next week.
Phone call 1 (before your quit day)	***A stop smoking advisor will give you a call*** on your agreed day and time. They will check that you have received your study product and answer any questions you may have on how to use it. They will then help you set a quit day and advise on preparing for it.
Phone calls 2-6 (on your quit day and 1-4 weeks after quitting)	During these ***weekly phone calls***, the advisor will provide support and guidance on quitting, and check on your progress. You will also be asked some questions about your product use and how you have been feeling.<br><br>You will be posted further supplies of your product as needed.
Phone call 7 (end of pregnancy)	***You will receive a phone call*** to complete a short questionnaire about your smoking, product use and health. If you have stopped smoking or reduced your smoking by over 50%, you will be sent a saliva sample kit and asked to return it back to us in the post. If you are required to do a saliva sample, we will send you a saliva kit with £10 and then once you return the sample and the site receive it you will be sent a further £10 for your time. If you are not smoking but still using a nicotine product, we will also ask you to attend an appointment to give a carbon monoxide reading. If you attend for this, you will be given £20 for your time.
Phone call 8 (3 months after having your baby)	***You will receive a phone call to*** find out about your smoking and you and your baby's health.

Who can take part?

You will be able to take part if you are:

- Aged 18 years or over
- 12-24 weeks pregnant
- A daily smoker wanting to quit
- Willing to use either EC or nicotine patches, with no strong preference for one or the other
- Willing to receive weekly and follow-up phone calls
- Able to speak English

You will **not** be able to take part if you:

- Have a known allergic reaction to nicotine skin patches (a contraindication for patch use)
- Are currently using NRT or EC daily
- Are taking part in another interventional trial (as per Good Clinical Practice)
- Have a serious medical problem or high-risk pregnancy

Benefits and Risks

We do not expect there to be any risks from using EC or nicotine patches to stop smoking. Nicotine patches are used routinely in pregnancy by the UK Stop Smoking Services. The most common side effect that people report experiencing when using patches is skin irritation.

EC do not contain tobacco, and therefore do not deliver the many harmful substances found in normal cigarettes. The vapour from EC contains propylene glycol which is approved for use in pregnancy (e.g. in asthma inhalers) and vegetable glycerol, which has no known adverse effects. Some flavourings may over time affect the user's lungs, but to a much smaller extent than smoking, and to our knowledge, no chemicals other than nicotine (which you would inhale anyway if you continued to smoke) have been identified in EC vapour that would be expected to affect the health of the baby. The most common side effects that people report experiencing when using EC are mouth/throat irritation. EC are not currently licensed as a medicine, but they are currently regulated as a consumer product.

The benefit of taking part in the study is that you will receive free, specialist stop-smoking treatment, which if successful, would not only improve your health but also that of your baby.

What if new information becomes available?

In the event of new information becoming available, you will be informed of this and will have the opportunity to withdraw from the study.

Data Protection

Queen Mary University of London is the sponsor for this study based in the United Kingdom. We will be using information from you in order to undertake this study and will act as the data controller for this study. This means that we are responsible for looking after your information and using it properly. Queen Mary University of London will keep identifiable information about you for 20 years after the study has finished.

Your rights to access change or move your information are limited, as we need to manage your information in specific ways in order for the research to be reliable and accurate. If you withdraw from the study, we will keep the information about you that we have already obtained. To safeguard your rights, we will use the minimum personally-identifiable information possible.

You can find out more about how we use your information at http://www.jrmo.org.uk/

The study team will collect information from you for this research study in accordance with our instructions.

Queen Mary University will use your name and contact details to contact you about the research study, and make sure that relevant information about the study is recorded for your care, and to oversee the quality of the study. Individuals from Barts Health NHS Trust and

regulatory organisations may look at your medical and research records to check the accuracy of the research study. Queen Mary University will pass these details to Barts Health NHS Trust along with the information collected from you. The only people in Barts Health NHS Trust who will have access to information that identifies you will be people who need to contact you for the purpose of the study or audit the data collection process. The people who analyse the information will not be able to identify you and will not be able to find out your name, or contact details.

You will be allocated a unique participant number by our database. Personal information will be stored on an electronic database created and held on a separate server to the anonymised participant data. Medical records will be accessed by research staff in order to collect safety data and your birth and maternal outcomes, but this data will be kept anonymised like the other data. We will inform your GP, with your consent, that you are taking part in this study. If you agree to it, we may use information held by the NHS and NHS Digital to keep in touch with you should we need to do longer-term follow-ups for safety outcomes. The results of this study may be presented to other individuals working in the field of smoking cessation or may be published in journals. However, all data will be anonymised and there will be no information included which could identify you.

What will happen to the samples I give?

The saliva samples you give will be anonymised like the rest of your data, and they will be sent via recorded delivery to the research unit (2 Stayner's Road, London E1 4AH), where they will be stored securely in a freezer for up to 3 years. At the end of the study, they will be sent to a laboratory (ABS Labs Ltd.) to be analysed. When the analysis is finished, the samples will be destroyed.

How have patients and the public been involved in this study?

We discussed e-cigarettes with our panel of smokers and with 4 women receiving treatment at our pregnancy stop-smoking service, and they influenced our decision to do this study. A panel of EC testers also tested EC and liquids for the study, and recommended which EC we should use. We plan to continue to involve patients and the public in the study by including at least 2 lay people in our Trial Steering Committee.

Your Rights

Your participation in this study is entirely voluntary, and you are free to drop out of the study at any time. Your records will be kept strictly confidential and your ordinary medical care will not be put at risk if you decide not to take part or drop out.

What happens if you are concerned or have any questions?

You will be able to contact Dr Katie Myers-Smith or Dr Dunja Przulj on 0207 882 8230 or via health-research@qmul.ac.uk if you are worried about anything or have any questions. The Chief Investigator of this study is Christopher Griffiths, Professor of Primary Care, Institute of Population Health Sciences, Yvonne Carter Building, 58 turner Street, London, E1 2AB, , Tel: 020 7882 2501.

A summary of the results of this study will be available upon request.

We believe that this study is safe and do not expect you to suffer any harm or injury because of your participation in it. However, Queen Mary University of London has agreed that if you are harmed as a result of your participation in the study, you will be compensated, provided that, on the balance of probabilities, an injury was caused as a direct result of the intervention or procedures you received during the course of the study. These special compensation arrangements apply where an injury is caused to you that would not have occurred if you were not in the trial. These arrangements do not affect your right to pursue a claim through legal action.

If you wish to raise a complaint or would like to seek independent advice outside the study team, you can call the local patient advice and liaison service (PALS) on 0203 594 2040/2050 or you can email them at pals@bartshealth.nhs.uk.

This study has been reviewed by the NRES Committee London South East.

This study is funded by the National Institute for Health Research Health Technology Assessment Programme (NIHR HTA Project 15/57/85).

We would like to thank you for your interest in this study.

NHS
National Institute for Health Research

Appendix 2

Schedule of follow-up calls

Text reminders were sent to participants the day before their follow up call was due. Participants were asked to text back if they did not wish to be called. Participants were also able to text back their smoking status if a call was not convenient. The follow-up efforts at EOP used the following protocol: 2 calls and text in the first week; 2 calls and text in second week; 1 call in week 3 followed by posting a questionnaire, emailing it and a text; no contact during weeks 4-5 to allow return of questionnaires; 2 calls in week 6; 1 text in week 7; 2 calls in week 8; 1 text in week 9; 1 call in week 10; 1 text in week 11 followed by an email; 1 call in week 12; 1 text in week 13; 1 call in week 14 and a final text in week 15. The follow-up efforts at 3 months PP: 2 calls and text in the first week; 1 call in second week followed by posting and emailing of questionnaire and a text; no contact during weeks 3-4 to allow return of questionnaires; 1 call and 1 text in week 5; final call in week 6.

Appendix 3: Study committees

**DMEC**	**Paul Aveyard (Chair)**
	Dominic Stringer (Statistician)
	Anne Greenough (Neonatologist)
**TSC**	**Jamie Brown (Chair)**
	Eleni Vangeli (expert in tobacco research)
	Leoni Brose (expert in tobacco research)
	Maryjane Winston (lay member)

Appendix 4: Summary of protocol amendments

Approved version*	Date	Summary
3.4	30/6/2017	Ethics committee recommended that age eligibility criteria be included in protocol (missed in error)
4.0	3/1/2018	Change in Sponsor representative; change to storage of paper forms
5.0	28/2/2019	Amendment to named statistician and change of name and address for CTU, data collection to include carbon monoxide measurement to verify abstinence in those using a nicotine product, replacement of participants randomised but later found ineligible, addition of new questions on respiratory health; addition of online survey method for follow up data collection; addition of collection of smoking status from hospital records at delivery
6.0	14/8/2019	Professor Christopher Griffiths added as new CI; update to saliva payments.
7.0	3/2/2020	Updates to protocol to reflect finalised SAP

*Versions prior to this were drafts before ethical and regulatory approvals.

Appendix 5: Leaflet with instructions on EC use

Helping Pregnant Smokers Quit: A Multi-Centre RCT of Electronic Cigarette and Nicotine Patches

[insert site logo]

How to use your electronic cigarette (EC)

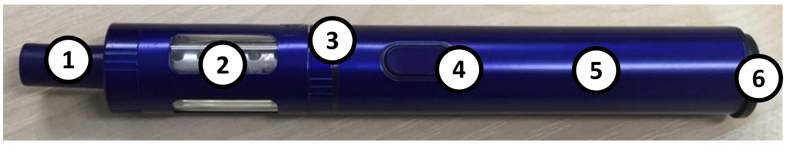

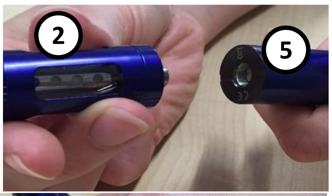

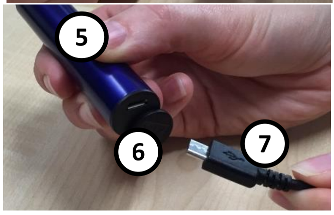

Charging your EC: Unscrew the battery (5) from the tank (2).

Twist the swivel cover (6) and connect the charger (7) to the port under the cover. The charger connects to a USB computer port, or to an adaptor so EC can be charged from a wall plug.

The EC needs about 30 minutes to charge. A red light on the charger will turn green when the battery is fully charged.

EC will need to be charged about once a day.

When your EC is charged, the button on the battery (4) glows green when pressed.

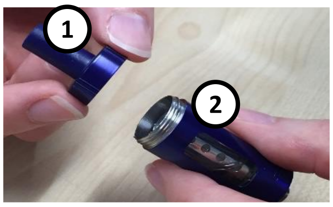

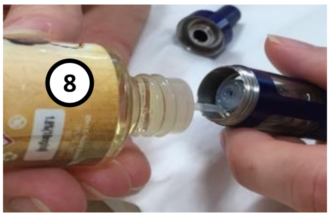

Filling your EC: Unscrew tank (2) from the battery (4), keep it facing up, and unscrew the mouthpiece (1). Place the mouthpiece on a piece of tissue.

Caps on e-liquid bottles (8) need to be pushed down before unscrewing them.

Insert the nozzle of the bottle into the tank aiming it at the side of the tank. Avoid getting any liquid into the central tube. Squeeze the bottle to fill the tank until it is almost full. Do not overfill.

Screw the mouthpiece and the tank back, do not overtighten the connections. EC will need refilling when the liquid has almost run out. When the coil is brand new, the liquid needs to soak into the coil for a few minutes.

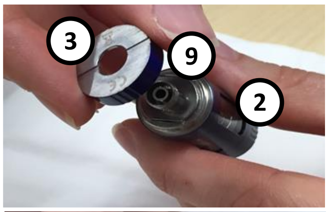

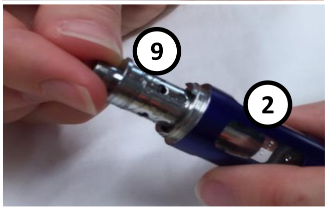

Replacing the coil: The tank (2) contains the coil (9) which is attached to the connector (3). The coil needs to be replaced about every 2 weeks. If the vapour starts to taste different it may be a sign that the coil needs changing.

With the tank empty and the battery unscrewed, unscrew the connector (3) and pull out the coil (9). You can wash the tank with hot water. When dry, insert a new coil making sure it aligns with the tank and then screw on the connector and battery.

General tips: * When using your EC for the first time, press the button (4) three times quickly to unlock it. (It will flash). To lock it, press it three times quickly again. (It will flash green when locked).
* If you get e-liquid on your skin, wipe and wash the area.
* Any condensation can be cleaned with a cotton bud, dirt in connections can be removed with a tooth pick. * As you hold down the button to vape, a little crackling sound is normal.

If you encounter any problems, call us on 0207 882 8230. *PREP EC instructions V3.1 27 April 2017*
